# VGLL3 Negatively Regulates Lipid Metabolism Genes FABP5 and FA2H, Modulating Inflammatory Responses in Systemic Lupus Erythematosus.

**DOI:** 10.51894/001c.143917

**Published:** 2025-09-30

**Authors:** Yilin Nie, Ruihao Lu, Yun Liang

**Affiliations:** 1 College of Osteopathic Medicine Michigan State University

## 18

### BACKGROUND

Systemic lupus erythematosus (SLE) is a chronic autoimmune disease with an unclear etiology. It is believed to arise from a complex interplay between genetic predisposition and environmental triggers. The underlying mechanism involves an aberrant immune response, where autoantibodies—most notably anti-nuclear antibodies—target the body’s own tissues, leading to widespread inflammation. Recent findings suggest that VGLL3, a transcription co-factor, may play a significant role in driving this bias by promoting immune activation and contributing to lupus pathogenesis. Our previous RNA-Seq study showed that Fabp5 (fatty acid-binding protein 5) and Fa2h (fatty acid 2-hydroxylase), genes implicated in lipid metabolism and inflammation, increased upon Vgll3 knockdown in the mouse skin.

### OBJECTIVES

We hypothesized that knockdown (KD) or knockout (KO) of Vgll3 would lead to elevated expression of Fabp5 and Fa2h, providing insight into their regulation and potential roles in inflammatory pathways.

Methods: To evaluate this hypothesis, we conducted VGLL3 knockdown experiments in human keratinocytes and Vgll3 knockout studies in mouse models. Western blotting and RT-qPCR were used to assess changes in protein and gene expression levels of Fabp5, Fa2h, and markers of inflammation, such as TNF-α.

### RESULTS

Our findings revealed that protein levels of FABP5 significantly increased following VGLL3 knockdown in human keratinocytes. Similarly, Fabp5 gene expression was markedly elevated in mouse skin following Vgll3 KO. Interestingly, TNF-α expression was downregulated in VGLL3-deficient human cells.

### CONCLUSION

These results suggest that Vgll3 negatively regulates Fabp5 expression, and its depletion reduces pro-inflammatory responses as reflected by lower TNF-α levels. These findings provide new insights into the regulatory network of genes downstream of Vgll3 and their contributions to inflammatory signaling in lupus pathogenesis. Further research is warranted to elucidate the mechanistic role of Fabp5 and Fa2h in interferon alpha treatment model.

